# Sustainable Epoxy Composites with UV Resistance Based on New Kraft Lignin Coatings

**DOI:** 10.3390/molecules29153697

**Published:** 2024-08-05

**Authors:** Rubén Seoane-Rivero, Patricia Ares-Elejoste, Koldo Gondra, Sara Amini, Pedro-Luis de Hoyos, Maria Gonzalez-Alriols

**Affiliations:** 1 GAIKER Technology Centre, Basque Research and Technology Alliance (BRTA), Parque Tecnológico de Bizkaia, Edificio 202, 48170 Zamudio, Spain; 2 Biorefinery Processes Research Group, Chemical & Environmental Engineering Department, Faculty of Engineering, Gipuzkoa, University of the Basque Country UPV/EHU, Plaza Europa 1, 20018 Donostia-San Sebastián, Spain

**Keywords:** sustainable, composite, epoxy, biobased, UV resistance, kraft lignin

## Abstract

Currently, the composite industry is focusing on more environmentally friendly resources in order to generate a new range of biobased materials. In this manuscript, we present a new work using lignocellulosic wastes from the paper industry to incorporate into biobased epoxy systems. The manufactured materials were composed of kraft lignin, glass fiber, and a sustainable epoxy system, obtaining a 40% biobased content. Using a vacuum infusion process, we fabricated the composites and analyzed their mechanical and UV resistance properties. The findings reveal a significant correlation between the lignin content and flexural modulus and strength, showing an increase of 69% in the flexural modulus and 134% in the flexural strength with the presence of 5% of lignin content. Moreover, it is necessary to highlight that the presence of synthesized lignin inhibits the UV degradation of the biobased epoxy coating. We propose that the use of lignocellulosic-based wastes could improve the mechanical properties and generate UV resistance in the composite materials.

## 1. Introduction

The need to develop materials in terms of strength, stability, lightness, and cost has been driven by industry and has generated rapid growth and strong demand from customers in different sectors, such as the automotive [1,2], wind [3,4], and train sectors [5]. Recently, there has also been an increased focus on the sustainability variable and that is why, nowadays, more and more researchers are working in biobased materials [6,7,8]. In addition to more sustainable resin systems, the use of renewable additives in composite materials has been considered in recent years, in order to improve properties and increase the biobased content. Composites are materials made from two or more constituent materials with significantly different physical or chemical properties. When combined, these constituents produce a material with characteristics different from the individual components [9]. The importance of composites in materials science lies in their ability to offer enhanced performance properties, such as higher strength, lighter weight, improved durability, and resistance to environmental degradation. These enhanced properties make composites critical in various applications, including the aerospace, automotive, and construction industries and for sporting goods [10].

Epoxy composites, a subset of this family of materials, are particularly significant due to their excellent mechanical properties and strong adhesive capabilities. They are formed by an epoxy resin matrix, which is usually reinforced with different types of additives [11]. The resulting material generally exhibits high strength-to-weight ratios, excellent thermal stability, and superior resistance to chemicals and moisture [12]. Throughout this work, we have worked with biobased gelcoat and epoxy resins. Although their price in comparison to other polymer systems are considerably higher, it is important to note their novelty and the renewable raw materials used for their synthesis. For example, the biobased gelcoat is formulated with 51% biobased carbons, reducing their carbon footprint without compromising performance. Moreover, it should be noted that they show very good properties compared to other resins, such as polyester resins. Traditionally, the additives incorporated into the epoxy matrix are derived from petrochemical sources, which are non-renewable and contribute to carbon emissions. Consequently, the introduction of biobased products into epoxy composites is essential to address the growing need for sustainable materials in response to environmental concerns [13]. In fact, by incorporating biobased materials, such as plant-derived oils, starches, or lignocellulosic components, the environmental impact can be significantly reduced. Moreover, these biobased materials offer several advantages, namely, a reduction in the carbon footprint, enhanced biodegradability, and resources renewability.

Lignocellulosic molecules, particularly lignin, play a crucial role in the development of sustainable epoxy composites. Lignin is a natural polymer found in the cell walls of plants and is a major byproduct of the paper and bioethanol industries [14]. Furthermore, its integration into epoxy composites offers several benefits, regarding the production process and the material properties. On the one hand, lignin is one of the most abundant organic polymers on Earth ensuring a readily available and sustainable raw material source, and it is relatively inexpensive, potentially reducing the overall cost of the composite production [15]. On the other hand, this natural polymer can enhance the mechanical strength and rigidity of epoxy composite materials. Furthermore, its aromatic structure contributes to better thermal stability of this type of composites, making them suitable for high-temperature applications [16]. Accordingly, in recent years, the incorporation of lignin into epoxy matrix-derived composites has attracted a great deal of attention. Thus, the synthesis of epoxy resins by reacting epichlorohydrin with lignin has been widely reported in the literature [17,18]. In this case, the lignin is an environmentally friendly source of hydroxyl groups, which can replace the use of other synthetic polymers such as bis phenol A (BPA). However, a common issue in the substitution of BPA with lignin can be its high polydispersity index, high molecular weight, and low solubility in some organic solvents and water [19]. To overcome this challenge, some studies have focused on the utilization of low-molecular weight lignin or lignin-derived oligomers [20,21]. In addition to that, another approach employed is the pretreatment of the lignin, i.e., its chemical modification. In this regard, various lignin modification reactions are implemented, e.g., phenolation [22], demethylation [23], esterification [24], glycidation [25], and amination [26].

Concerning the properties of the resulting epoxy–lignin composites, once the previously mentioned hurdles are solved, it can be confirmed in general that the incorporation of lignin results in epoxy composites with enhanced mechanical and thermal properties. In a recent study by Xue et al. 2021, the elaboration of epoxy composites with different degrees of substitution of BPA with lignin was presented, and they showed the mentioned improved tendency regarding the mechanical performance of these materials. Thus, they found that the composites with 100% of lignin were the ones showing the highest values for tensile strength [27]. Another work by Zhen et al. 2021 proved that the introduction of lignin not only achieved higher values for flexural strength and storage modulus but also promoted a certain degree of flame retardancy in the resins [28]. In addition to yielding some level of fireproofing, lignin added into the epoxy composites produced an increment in the thermal properties as well. Thereby, it has recently been reported by X. Wang et al. [29] that an increase in the amount of lignin in different epoxy resin formulations leads to a higher percentage of residue after thermogravimetric analysis. Likewise, they showed that the formulation with the higher amount of lignin was the one with the highest value of T50%. In this work, a biobased epoxy gelcoat was combined with lignin in order to fabricate new sustainable composites with UV resistance capabilities. We synthesized new kraft lignin from the Zicuñaga pulp and paper industry (Gipuzkoa, Spain), which was integrated into the coating of the composite. Glass fiber was used as reinforcement and epoxy resin with 38% of biobased content was used in order to manufacture the composite by an infusion process.

## 2. Results

The KL sample’s characterization by a GPC analysis indicated that the lignin presented a molecular weight (Mw) of 4936 g/mol and a polydispersity value (Mw/Mn) of 4102, which means that the lignin presented a medium molecular weight and polydispersity value, suitable for being used in a wide variety of formulations [30].

The characterization of the KL samples in terms of the hydroxyl groups’ content, conducted with a 31P NMR spectroscopy, presented the results included in Table 1. The concentrations of the OH groups in aliphatic, C5-substituted, guaiacyl, p-hydroxyphenyl, and carboxylic acid forms were measured (mmol OH/g lignin units). The syringyl/guaiacyl (S/G) monomers’ ratio was also calculated by dividing the OH groups of C5 substitutes (considered as the syringyl OH groups as a result of signal overlapping) into guayacyl OH groups. This ratio provides information about the number of β-O-4 bonds present in the lignin network. The formation of a monomeric unit requires the cleavage of two β-O-4 bonds, one on each side of the aromatic moiety. This means that, during the lignin biosynthesis, G units tend to form C–C bonds, resulting in a decrease in the formation of the ether bonds that generate monomers.

The obtained results are in good concordance with the biomass source (hardwood), due to the low content of p-hydroxyphenyl units, which appear mainly in coniferous wood types and herbaceous species. The value of the S/G ratio, much bigger than one, also corresponded to a hardwood species [31]. The results of the elemental analysis of the KL sample and SGi128 + KL (5% wt.) coating sample are included in Table 2.

**Figure 1:**
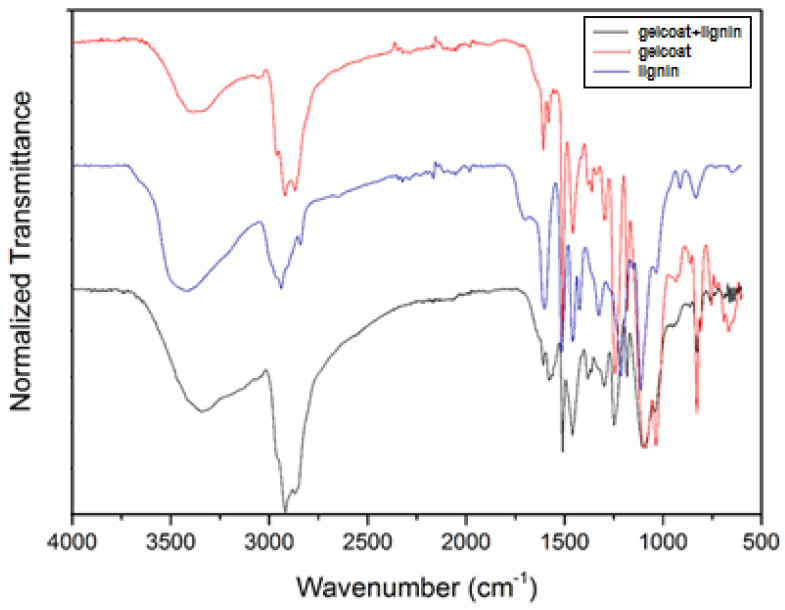
Infrared spectra obtained by FTIR analysis.

The results of the elemental composition reveal a high nitrogen percentage provided by the coating formulation. The FTIR spectra of the KL (5% wt.) sample, SGi128 coating sample, and KL (5% wt.) + SGi128 coating sample are presented in Figure 1.

The main bands identified in the fingerprint region are classified in Table 3.

The bands related to the G and S structures are coherent with the raw material type (hardwood), and the spectrum of the lignin plus gelcoat shows a good integration of lignin in the matrix.

DSC tests were carried out to characterize the thermal transitions of the polymeric system and the effect of the lignin in the gelcoat. The obtained results can be found in Table 4. In the analyzed systems, in the first run from an ambient temperature to 280 °C, an exothermic peak with a maximum exothermic temperature was obtained, due to the curing reaction of both epoxy systems.

**Figure 2:**
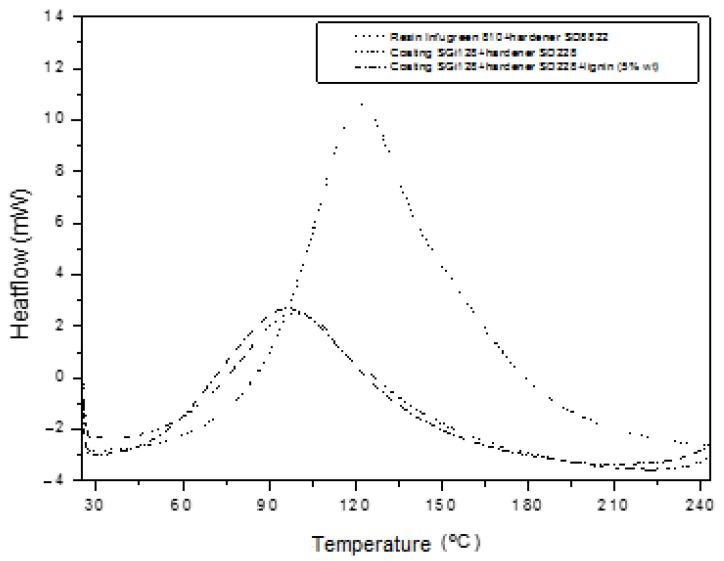
Thermal properties of polymer systems.

All the polymer systems showed a single T_max_ on the DSC measurements, as can be seen in Figure 2. It is important to note that these exothermic peaks were between approximately 97 °C and 120 °C. Once the lignin was introduced, the exothermic temperature decreased by around 20 °C. Therefore, it can be considered that lignin has no effect on either the exothermic temperature or the curing process. This result is expected, because there are some studies that demonstrate that lignin has little impact on thermal properties [34,35].

**Figure 3:**
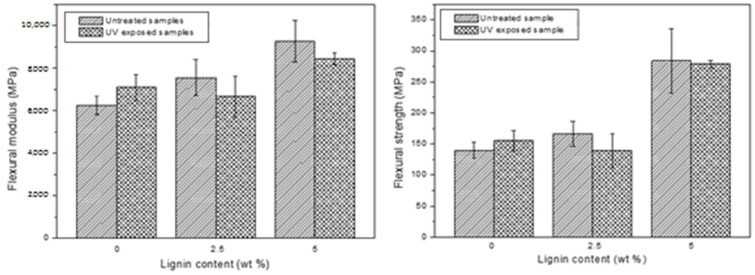
Mechanical properties of composites.

The mechanical properties of the manufactured composites are shown in Figure 3. As can be seen in the aforementioned figure, lignin gives better mechanical properties to the composites’ structures. Moreover, as the lignin content increases, the flexural strength and modulus also improve significantly. The results confirm that the flexural modulus increases around 31% with the presence of 2.5% wt. lignin content and 69% with 5% wt. Regarding the flexural strength, this effect is more noticeable, as it increases around 34% with the presence of 2.5% wt. lignin content and 134% with 5% wt. This fact shows that the introduction of this biomass additive has a significant improvement on the flexural properties. This can be an indicator of the reinforcement effect of lignin in the biobased gelcoat. Not much information is found in the literature on the mechanical properties of thermoset composites containing lignin, but, for example, Wu Zhe et. al. analyzed how the addition of lignin fiber changed the compression characteristics of the matrix material, resulting in the appearance of a strengthening stage [36].

**Figure 4:**
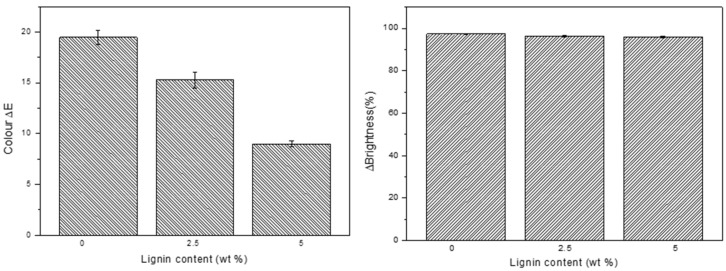
Color (**left**) and brightness (**right**) variation of fabricated composites.

Regarding the UV effect on the mechanical properties, it can be said that the samples did not suffer high changes after degradation, because this attack is focused on the gelcoat. It was observed that the samples without lignin increased their modulus and strength, whereas the samples with this biofiller at a 5% wt. content showed higher values of these parameters after UV irradiation, as observed in previous studies [37]. It is remarkable that the mechanical properties of the KL (5% wt.) samples reported better values than the ones that did not contain lignin and did not suffer the UV attack. With the goal of characterizing the UV resistance capability of the manufactured composites in the presence of lignin content, the brightness and color were analyzed. Figure 4 shows the brightness variation and color changes.

As can be seen, the effect of UV attack and water condensation at 50 °C is more significant in the color than in the brightness properties. This may be because the samples were matt and white in color.

Moreover, it is important to note that the color variation decreases with the lignin content, so it can be concluded that the effect of lignin is considerable in UV resistance, decreasing the ΔE (color) by more than 10 between the reference and the composite with KL (5% wt.).

**Figure 5:**
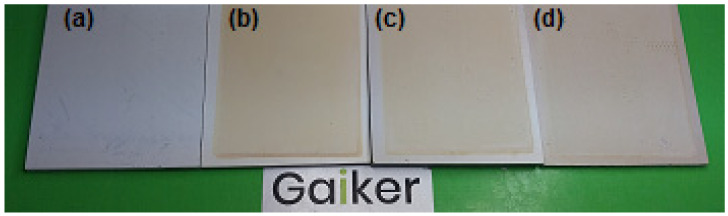
(**a**) Reference; (**b**) reference after UV treatment; (**c**) KL (2.5% wt.) sample after UV treatment; and (**d**) KL (5% wt.) sample after UV treatment.

As can be observed in Figure 5, the color changes are visible to the naked eye, due to the yellowing caused by UV radiation. It should be taken into account that there is a notable difference in the color between the sample without lignin content and the sample that contains 5% wt. In order to characterize the effect of UV treatment on surface roughness, a confocal microscopy was also used. The surface topography measurements were conducted before and after the UV attack.

**Figure 6:**
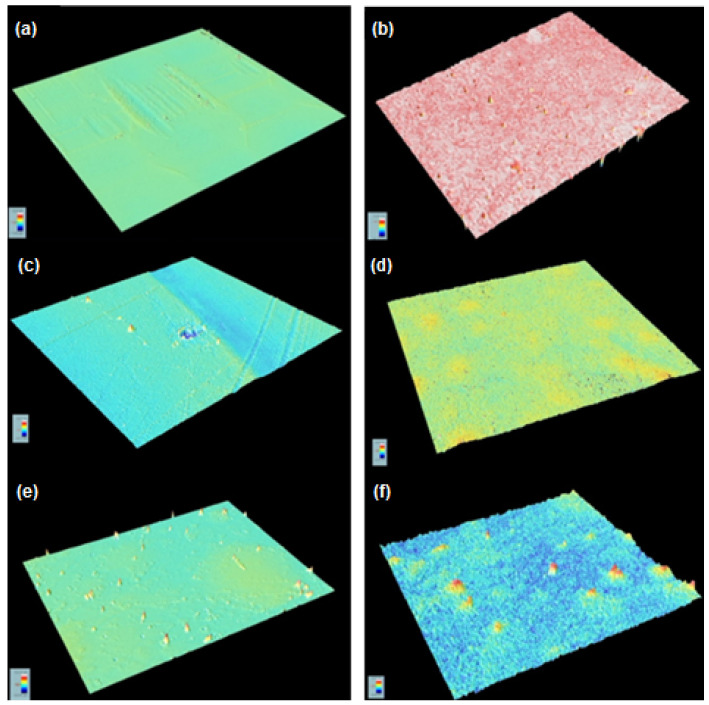
(**a**) Reference; (**b**) reference after UV treatment; (**c**) KL (2.5% wt.) sample; (**d**) KL (2.5% wt.) sample after UV treatment; (**e**) KL (5% wt.) sample; and (**f**) KL (5% wt.) sample after UV treatment.

The samples that did not undergo the UV attack showed a similar roughness, with Sa values of around 1.6 μm, as can be seen in Figure 6. In contrast, after suffering water condensation at 50 °C and UV radiation, the samples increased their roughness considerably; in the case of the reference, the roughness increased around 171%, while the samples that contained lignin showed a percentage lower: 157% for the KL (2.5% wt.) sample and 132% in the case of the KL (5% wt.) sample, as can be seen in Table 5. The photodegradation effect was higher in the samples with less lignin content.

## 3. Materials and Methods

### 3.1. Epoxy Resin System

A commercial epoxy resin, InfuGreen 810, with 38% of biobased content (a carbon of plant origin) was used. The hardener that was used was SD 822, supplied by Sicomin Epoxy Systems (France). This system shows a very low viscosity at room temperature, and it can be mixed with different hardeners. The most relevant physicochemical properties of the above-mentioned system are shown in Table 6.

The applied coating was also supplied by Sicomn Epoxy Systems and was formulated with 51% of the biobased gelcoat called SGi 128 combined with the SD 228 hardener. This is a halogen-free and fire-resistant epoxy coating with low levels of smoke density and gas toxicity. The reinforcement used was a quadriaxial glass fiber with a grammage of 450 g/m^2^.

### 3.2. Kraft Lignin

Kraft lignin (KL) was precipitated from black liquors provided by the Zicuñaga pulp and paper industry (Gipuzkoa, Spain). This industry processes eucalyptus wood. Kraft lignin is considered a medium–high-molecular weight lignin with values ranging from 200 to 200,000 g/mol, high carbohydrate contents due to condensation reactions during the kraft process, and 1–3% sulfur contents [38]. KL is precipitated by the addition of acid, protonating the phenolic compounds present in lignin, lowering their hydrofiliation, and precipitating as a result. In this case, precipitation was carried out by acidifying the liquor to a pH of 4 using glacial acetic acid. Once the lignin precipitation was completed, the solution was left to stand for 24 h. Then, a vacuum filtration was performed, and the lignin sample recovered in the filtrate, which was washed with distilled water until it was neutralized. Finally, the recovered lignin was dried for 2 h at 28 °C and stored.

### 3.3. Kraft Lignin Characterization

The lignin samples were characterized by several techniques. A gel permeation chromatography/size exclusion chromatography (GPC) was used to determine the average molecular weight and size distribution of the lignins. A Jasco Inc. chromatograph with an LC-NettII/ACD interface, a CO-2065Plus oven column, and an RI-2031PlusIntelligent Refractive Index detector was used. In addition, the chromatograph was equipped with a guard column and two PolarGel-M columns (Varian Inc., Palo Alto, CA, USA) in series. DMF was used as the mobile phase at a flow rate of 0.7 mL/min and a temperature of 40 °C. The instrument was calibrated with polystyrene standards in the molecular weight range of 250–70,000 g/mol. For the analysis, 25–50 mg of the sample was taken and dissolved in 5 mL of the mobile phase.

One of the most important properties of lignin is the content of its hydroxyl compounds, as it acts on solubility and reactivity. Consequently, the quantification of the different OH groups of the molecules is of great importance. ^31^P NMR is a quantitative determination by spectroscopy. Phosphorylation was carried out followed by phosphorus nuclear magnetic resonance (^31^P NMR) spectroscopy in Bruker AVANCE II 400 equipment to analyze the content of hydroxyl groups in the lignin samples. 

An elemental analysis was performed using a Leco TruSpec microanalyzer at 1050 °C. Both the carrier gas (3X pure helium) and test gas (4X extra-pure oxygen) were supplied by Nippon Gases. The calibration was performed using Leco Sulfamethanize (C = 51.78%; H = 5.07%; and S = 11.5%). The samples were analyzed in triplicate.

To evaluate the main structural level characteristics and functional groups of lignin, the characterization was performed with Fourier transform infrared (FTIR) spectroscopy using a PerkinElmer Spectrum Two FT-IR instrument equipped with an attenuated total reflectance (ATR) accessory and an internally reflecting diamond glass lens. The samples were analyzed using 64 scans with a resolution of 4 cm^−1^ and a wave number range of 4000–400 cm^−1^.

### 3.4. Biobased Resin System and Gelcoat Formulation Characterization

To determine the reactivity of the resin system before using it in the infusion process, a thermal analysis was performed. In the same manner, the influence of the lignin on the reactivity of the gelcoat system was studied in order to prevent it from inhibiting the curing at a concentration of 5% wt.

The thermal transitions of the resin and gelcoat were analyzed by a differential scanning calorimetry (DSC) on a Mettler Toledo (DSC 822e) calorimeter equipped with a liquid nitrogen accessory. The samples were weighed on a METTLER TG 50 4-digit analytical balance in 40 µL aluminum pans. These samples were weighed in the liquid state (approximately 10 mg), in order to obtain the maximum exothermic temperature peak, T_max_, and the heat of the reaction. With regard to the operating conditions, the samples were heated from 25 °C to 280 °C at a rate of 10 °C·min^−1^ and under an inert nitrogen atmosphere. The data in Table 4 were taken from the first scan.

Once the formulation was cured, to observe the effect of lignin on the systems, especially at the level of the elemental composition and morphology, a scanning electron microscopy (SEM), using a Zeis EVO 50 microscope at 20 kV and an energy dispersive X-ray (EDX) (INCA, Oxford Instruments, Oxford, United Kingdom), was conducted. The cured formulations were coated by a sputter Leica EM SCD005, before the measurements were conducted with gold palladium. Further, a 3D optical profiler PLμ NEOX (Sensofar) was used for the surface topography measurements.

### 3.5. Composite Development and Characterization

**Figure 7:**
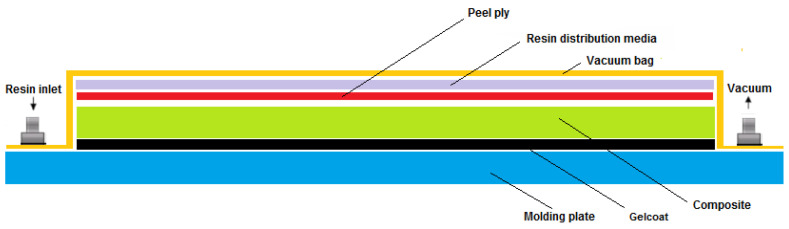
Scheme of the vacuum infusion technique.

The biobased epoxy composites were developed using a vacuum infusion process. This process provides high-performance composites for different sectors such as the wind, aeronautic, automotive, and railway sectors. The vacuum infusion technique is as represented in Figure 7.

Firstly, the release agent was applied on the substrate (the molding plate), in order to facilitate the subsequent demolding of the composite. Then, a layer of gelcoat (SGi 128 combined with the SD228 hardener) and different percentages of lignin (2.5 and 5% wt.) were deposited on the glass substrate and, after 4 h, when the gelcoat was in a tacky state, five layers of fiberglass were stacked. Once stacked, a peel ply was placed to facilitate the demolding of the material after the process. A distribution mesh was then placed to help to achieve a correct and homogeneous impregnation of the fiber. Next, two tubes were placed in the system, one for the vacuum and the other for the resin injection, and then the system was sealed with a film (comprising the counter-mold). After assembling the system, the resin was injected until the fiber was properly impregnated and cured, according to the manufacturer’s curing and post-curing conditions.

After the composite was manufactured, several tests, such as mechanical tests, a UV attack, and color and brightness characterizations, were carried out.

The mechanical properties were focused on the flexural tests. These tests were performed in accordance with ISO 14125:1998/A1:2011 [39]. A support spacing of 50 mm and a test speed of 1 mm/min were used. Samples of dimensions 15 × 2.9 mm were prepared and loaded to failure. The measurements were carried out with a load cell of 5 kN. For each composite, three samples were tested and the average value was reported.

The UV attack was performed according to UNE EN ISO 4892-3:2016 (Method A) [40], which requires a total time of 500 h. The samples were subjected to cycles of 8 h of UV exposure at 70 °C with 340 nm UVA lamps, followed by 4 h of water condensation at 50 °C.

The color measurements were performed according to UNE EN ISO 11664-1:2011 [41]. After the measurement, the colorimeter recorded the L, a, and b parameters. To obtain the color variation as expressed in the results section, a series of transformations detailed in the standards were used. Moreover, gloss measurements were performed according to the UNE-EN ISO 2813:1999 standard [42]. This method uses three angles of incidence of the light beam: 20°, 60°, and 85°. For simplification, the measurements obtained with the 60° angle were compared. An elemental analysis of the composite was performed using the equipment and conditions indicated in the lignin characterization section.

## 4. Conclusions

Nowadays, it can be said that there is interest from the composites industry in obtaining new sustainable alternatives to decrease the environmental impact of composite materials. This manuscript introduces the concept of using lignocellulosic-based wastes from the paper industry and, on the other hand, the incorporation of biobased epoxy systems (resin and gelcoat), in order to generate new sustainable epoxy composites.

In this manuscript, we present a work using a new kraft lignin from the Zicuñaga pulp and paper industry (Gipuzkoa, Spain), which was incorporated into a biobased epoxy system. Firstly, through a GPC, elemental analysis, and RMN characterization, we ensured that the synthesis was in good concordance with the biomass source. Secondly, it is important to note the significant improvement of the mechanical properties after introducing synthetized lignin in the coating of the composite. These results show that the presence of 5% of lignin content increased the flexural modulus by 69% and the flexural strength by 134%. The UV resistance was also characterized by color and brightness tests and a confocal microscopy. While the brightness test did not show any difference between the reference and samples with 2.5 and 5% of lignin content, the color and roughness parameters demonstrated that the presence of lignin inhibited the UV degradation of the biobased epoxy coating. Considering the presented arguments and research findings, it can be concluded that we demonstrate that the use of lignocellulosic-based wastes could make new properties in the composites’ coatings, improving their mechanical properties and generating UV resistance.

## Figures and Tables

**Table 1:** Content of different hydroxyl groups in KL samples (mmol/g lignin) obtained after RNM analysis.

Aliphatic OH	C5-Substituted OH	Guaiacyl OH	p-Hydroxyphenyl OH	Carboxylic Acid OH	S/G Ratio
2.04	4.63	0.28	1.56	0.28	16.38

**Table 2:** Results of the elemental analysis.

Sample	% C	% H	% N	% S	% O (by Difference)
KL (5% wt.)	61.63	5.63	0.13	6.60	26.01
KL + SGi128	41.11	6.16	14.15	2.56	36.02

**Table 3:** Identification of the bands in the fingerprint region from the kraft lignin FTIR [32,33].

Wavenumber (cm^−1^)	Band Assignment
3450	Stretching of the aliphatic O–H bond
2934, 2831	C–H stretch in methyl and methylene groups
1600	Aromatic skeleton vibrations plus C–O stretching
1510	Aromatic vibrations of G units
1574	Aromatic skeleton vibrations plus C–O stretching
1458	C–H deformations (asymmetric in –CH_3_ and –CH_2_–)
1423	Aromatic skeleton vibrations combined with C–H in plane deformations
1383	Aliphatic C–H stretching in CH_3_ and phenolic OH
1363	Aliphatic C–H stretching in CH_3_ and phenolic OH
1299	G ring plus C+O stretching
1245	C–C, C–O, and C=O stretching
1183	Typical for HGS lignin samples; C–O in ester groups
1099	Aromatic C–H deformation of S units
1038	Aromatic C–H in plane deformation plus C–O deformation in primary alcohols plus C–H stretching (unconjugated)
911	C–H out of plane (aromatic rings)
827	C–H out of plane in positions 2, 5, and 6 (G units)

**Table 4:** Thermal properties of different epoxy systems.

Formulation	Tm (°C)	ΔH (J/g)
Infugreen 810 resin + SD8822 hardener	122	496
SGi128 coating + SD228 hardener	99	225
SGi128 coating + SD228 hardener + lignin (5% wt.)	97	210

**Table 5:** Surface roughness values of reference, KL (2.5% wt.) sample, and KL (5% wt.) sample before and after the UV radiation.

	Sa (μm)		
	Reference	KL (2.5% wt.)	KL (5% wt.)
**Initial**	1.6 ± 0.1	1.8 ± 0.2	1.4 ± 0.1
**500 h UV**	4.4 ± 0.3	4.6 ± 0.3	3.4 ± 0.2

**Table 6:** Physicochemical properties of the epoxy resin used.

Property	Units	Value
Viscosity at 20 °C	mPas	215.0
Density at 20 °C	g/cm^3^	1.2
Pot life at 20 °C 500 g.	h	4.7

## Data Availability

The data are available in this manuscript.

## References

[1] Matta A., Yadavalli V.R., Manas L., Kadleckova M., Pavlinek V., Sedlacek T. (2024). Surface Treatments’ Influence on the Interfacial Bonding between Glass Fibre Reinforced Elium^®^ Composite and Polybutylene Terephthalate. Materials.

[2] Capretti M., Del Bianco G., Giammaria V., Boria S. (2024). Natural Fibre and Hybrid Composite Thin-Walled Structures for Automotive Crashworthiness: A Review. Materials.

[3] Bulińska S., Sujak A., Pyzalski M. (2024). From Waste to Renewables: Challenges and Opportunities in Recycling Glass Fibre Composite Products from Wind Turbine Blades for Sustainable Cement Production. Sustainability.

[4] Broniewicz M., Halicka A., Buda-Ożóg L., Broniewicz F., Nykiel D., Jabłoński Ł. (2024). The Use of Wind Turbine Blades to Build Road Noise Barriers as an Example of a Circular Economy Model. Materials.

[5] Diniță A., Ripeanu R.G., Ilincă C.N., Cursaru D., Matei D., Naim R.I., Tănase M., Portoacă A.I. (2024). Advancements in Fiber-Reinforced Polymer Composites: A Comprehensive Analysis. Polymers.

[6] Capretti M., Giammaria V., Santulli C., Boria S., Del Bianco G. (2023). Use of Bio-Epoxies and Their Effect on the Performance of Polymer Composites: A Critical Review. Polymers.

[7] Samaniego-Aguilar K., Sánchez-Safont E., Rodríguez A., Marín A., Candal M.V., Cabedo L., Gamez-Perez J. (2023). Valorization of Agricultural Waste Lignocellulosic Fibers for Poly(3-Hydroxybutyrate-Co-Valerate)-Based Composites in Short Shelf-Life Applications. Polymers.

[8] Hiremath P., Ranjan R., DeSouza V., Bhat R., Patil S., Maddodi B., Shivamurthy B., Perez T.C., Naik N. (2023). Enhanced Wear Resistance in Carbon Nanotube-Filled Bio-Epoxy Composites: A Comprehensive Analysis via Scanning Electron Microscopy and Atomic Force Microscopy. J. Compos. Sci..

[9] Atmakuri A., Palevicius A., Vilkauskas A., Janusas G. (2020). Review of hybrid fiber based composites with nano particles—Material properties and applications. Polymers.

[10] Oladele I.O., Omotosho T.F., Adediran A.A. (2020). Polymer-based composites: An indispensable material for present and future applications. Int. J. Polym. Sci..

[11] Peerzada M., Abbasi S., Lau K.T., Hameed N. (2020). Additive manufacturing of epoxy resins: Materials, methods, and latest trends. Ind. Eng. Chem. Res..

[12] Pappa C., Feghali E., Vanbroekhoven K., Triantafyllidis K.S. (2022). Recent advances in epoxy resins and composites derived from lignin and related bio-oils. Curr. Opin. Green Sustain. Chem..

[13] Gonçalves F.A., Santos M., Cernadas T., Ferreira P., Alves P. (2022). Advances in the development of biobased epoxy resins: Insight into more sustainable materials and future applications. Int. Mater. Rev..

[14] Sharma S., Sharma A., Mulla S.I., Pant D., Sharma T., Kumar A. (2020). Lignin as potent industrial biopolymer: An introduction. Lignin: Biosynthesis and Transformation for Industrial Applications.

[15] Agustiany E.A., Rasyidur Ridho M., Rahmi DN M., Madyaratri E.W., Falah F., Lubis M.A.R., Solihat N.N., Syamani F.A., Karungamye P., Sohail A. (2022). Recent developments in lignin modification and its application in lignin-based green composites: A review. Polym. Compos..

[16] Li C., Wang B., Zhou L., Hou X., Su S. (2022). Effects of lignin-based flame retardants on flame-retardancy and insulation performances of epoxy resin composites. Iran. Polym. J..

[17] Gouveia J.R., Garcia G.E., Antonino L.D., Tavares L.B., Dos Santos D.J. (2020). Epoxidation of kraft lignin as a tool for improving the mechanical properties of epoxy adhesive. Molecules.

[18] Li W.X., Xiao L.P., Li X.Y., Xiao W.Z., Yang Y.Q., Sun R.C. (2021). Renewable and flexible thermosetting epoxies based on functionalized biorefinery lignin fractions. Mater. Today Sustain..

[19] Zhao S., Abu-Omar M.M. (2017). Synthesis of renewable thermoset polymers through successive lignin modification using lignin-derived phenols. ACS Sustain. Chem. Eng..

[20] Liu G., Jin C., Huo S., Kong Z., Chu F. (2021). Preparation and properties of novel bio-based epoxy resin thermosets from lignin oligomers and cardanol. Int. J. Biol. Macromol..

[21] Nicastro K.H., Kloxin C.J., Epps T.H. (2018). Potential lignin-derived alternatives to bisphenol A in diamine-hardened epoxy resins. ACS Sustain. Chem. Eng..

[22] Zhen X., Li H., Xu Z., Wang Q., Zhu S., Wang Z., Yuan Z. (2021). Facile synthesis of lignin-based epoxy resins with excellent thermal-mechanical performance. Int. J. Biol. Macromol..

[23] Zhang S., Zhao X., Chen P., Sun G., Li Y., Han Y., Wang X., Li J. (2023). High-performance adhesives modified by demethylated lignin for use in extreme environments. New J. Chem..

[24] Chen B., Zhang Q., Lu M., Meng H., Qu Z., Xu C.A., Jiao E. (2021). Synthesis of a novel lignin-based epoxy resin curing agent and study of cure kinetics, thermal, and mechanical properties. J. Appl. Polym. Sci..

[25] Over L.C., Grau E., Grelier S., Meier M.A., Cramail H. (2017). Synthesis and characterization of epoxy thermosetting polymers from glycidylated organosolv lignin and bisphenol A. Macromol. Chem. Phys..

[26] Pan H., Sun G., Zhao T., Wang G. (2015). Thermal properties of epoxy resins crosslinked by an aminated lignin. Polym. Eng. Sci..

[27] Xue B., Tang R., Xue D., Guan Y., Sun Y., Zhao W., Tan J., Li X. (2021). Sustainable alternative for bisphenol A epoxy resin high-performance and recyclable lignin-based epoxy vitrimers. Ind. Crops Prod..

[28] Zhen X., Li H., Xu Z., Wang Q., Xu J., Zhu S., Yuan Z. (2021). Demethylation, phenolation, and depolymerization of lignin for the synthesis of lignin-based epoxy resin via a one-pot strategy. Ind. Crops Prod..

[29] Wang X., Leng W., Nayanathara R.O., Caldona E.B., Liu L., Chen L., Zhang X. (2022). Anticorrosive epoxy coatings from direct epoxidation of bioethanol fractionated lignin. Int. J. Biol. Macromol..

[30] Li T., Takkellapati S. (2018). The current and emerging sources of technical lignins and their applications. Biofuels Bioprod. Biorefining.

[31] Del Río J.C., Gutiérrez A., Hernando M., Landín P., Romero J., Martínez Á.T. (2005). Determining the influence of eucalypt lignin composition in paper pulp yield using Py-GC/MS. J. Anal. Appl. Pyrolysis.

[32] Anderson E.M. (2019). Differences in S/G ratio in natural poplar variants do not predict catalytic depolymerization monomer yields. Nat. Commun..

[33] Chen L. (2015). Study on pyrolysis behaviors of non-woody lignins with TG-FTIR and Py-GC/MS. J. Anal. Appl. Pyrolysis.

[34] Barzegari M.R., Alemdar A., Zhang Y., Rodrigue D. (2013). Thermal Analysis of Highly Filled Composites of Polystyrene with Lignin. Polym. Polym. Compos..

[35] Nair S.S., Kuo P.Y., Chen H., Yan N. (2017). Investigating the effect of lignin on the mechanical, thermal, and barrier properties of cellulose nanofibril reinforced epoxy composite. Ind. Crops Prod..

[36] Zhe W., Qingnan W., Shuai Z., Yang Z., Bo X. (2013). Study on Mechanical Properties and Fracture Mechanisms of Lignin Fiber/epoxy Resin Composites. Mater. Plast..

[37] Wang H., Lin W., Qiu X., Fu F., Zhong R., Liu W., Yang D. (2018). In Situ Synthesis of Flowerlike Lignin/ZnO Composite with Excellent UV-Absorption Properties and Its Application in Polyurethane. ACS Sustain. Chem. Eng..

[38] Lora J.H., Glasser W.G. (2002). Recent industrial applications of lignin: A sustainable alternative to non-renewable materials. J. Polym. Environ..

[39] (2002). Fibre-Reinforced Plastic Composites—Determination of Flexural Properties.

[40] (2016). Plastics—Methods of Exposure to Laboratory Light Sources—Part 3: Fluorescent UV Lamps.

[41] (2011). Colorimetry—Part 1: CIE Standard Colorimetric Observers.

[42] (2015). Paints and Varnishes—Determination of Gloss Value at 20º, 60º and 85º (ISO 2813:2014).

